# Bivalent promoter marks and a latent enhancer may prime the leukaemia oncogene *LMO1* for ectopic expression in T-cell leukaemia

**DOI:** 10.1038/leu.2013.2

**Published:** 2013-02-01

**Authors:** S H Oram, J Thoms, J I Sive, F J Calero-Nieto, S J Kinston, J Schütte, K Knezevic, R B Lock, J E Pimanda, B Göttgens

**Affiliations:** 1 Department of Haematology, Cambridge Institute for Medical Research and Wellcome Trust and MRC Cambridge Stem Cell Institute, University of Cambridge, Cambridge, UK; 2 Lowy Cancer Research Centre and The Prince of Wales Clinical School, University of New South Wales, Sydney, New South Wales, Australia

**Keywords:** *LMO1*, transcriptional regulation, T-ALL, bivalent chromatin

## Abstract

*LMO1* is a transcriptional regulator and a T-acute lymphoblastic leukaemia (T-ALL) oncogene. Although first identified in association with a chromosomal translocation in T-ALL, the ectopic expression of *LMO1* occurs far more frequently in the absence of any known mutation involving its locus. Given that *LMO1* is barely expressed in any haematopoietic lineage, and activation of transcriptional drivers in leukaemic cells is not well described, we investigated the regulation of this gene in normal haematopoietic and leukaemic cells. We show that *LMO1* has two promoters that drive reporter gene expression in transgenic mice to neural tissues known to express endogenous *LMO1*. The *LMO1* promoters display bivalent histone marks in multiple blood lineages including T-cells, and a 3' flanking region at *LMO1* +57 contains a transcriptional enhancer that is active in developing blood cells in transgenic mouse embryos. The *LMO1* promoters become activated in T-ALL together with the 3' enhancer, which is bound in primary T-ALL cells by *SCL*/TAL1 and *GATA3*. Taken together, our results show that *LMO1* is poised for expression in normal progenitors, where activation of *SCL*/TAL1 together with a breakdown of epigenetic repression of *LMO1* regulatory elements induces ectopic *LMO1* expression that contributes to the development and maintenance of T-ALL.

## Introduction

Lim-only 1 (*LMO1*) also known as T-cell translocation gene 1 (*TTG-1*) and Rhombotin 1 (*RBTN-1*) encodes a LIM domain transcriptional cofactor, and was originally identified at a chromosomal breakpoint in a T-acute lymphoblastic leukaemia (T-ALL) cell line bearing the t(11;14)(p15;q11) translocation.^1, 2^ *LMO1* is not normally expressed in T-cells, but the t(11;14)(p15;q11) juxtaposes *LMO1* to gene regulatory elements within the T-cell receptor α/δ loci leading to ectopic expression in a T-lymphoid environment. Even though <1% of T-ALL patients carry translocations involving *LMO1*, ∼50% express significant levels of *LMO1*,^3^ thus suggesting that aberrant activation of regulatory elements within the *LMO1* gene locus represents a common feature of dysregulated transcriptional programs in T-ALL. Given the unequivocal demonstration through transgenic mouse experiments that ectopic *LMO1* expression in T-cells causes T-ALL,^4^ a detailed understanding of transcriptional control mechanisms operating at the *LMO1* gene locus would appear vital to elucidate the mechanisms responsible for ectopic expression in T-ALL patients without *LMO1* translocations.

Despite its long-established role as a T-ALL oncogene, relatively little is known about the normal function of *LMO1*. *Lmo1*-homozygous null mice are viable with no discernible phenotype.^5^ As knockout mice for the highly related *Lmo3* gene were similarly viable^5^ and because the expression domains of *Lmo1* and *Lmo3* overlap,^6^ *Lmo1* and *Lmo3* double knockout mice were also generated, which lead to perinatal death, the cause of which was not determined.^5^ When expressed ectopically in T-cells in transgenic mice together with *SCL*/TAL1, double-transgenic mice display abnormalities of thymocyte development with altered proliferation, apoptosis and immunophenotype prior to the onset of a frank T-cell malignancy.^7^ LMO1 forms multiprotein complexes with transcriptional regulators such as SCL,^8, 9^ LDB1,^10^ GATA3.^11^ LMO1 has also been proposed to participate in a Hox-dependent regulatory network in the developing hindbrain.^12^ However, no significance during normal LMO1 function has as yet been ascribed to the formation of such protein complexes. Finally, there has been considerable recent interest in the role of LMO1 in neurological malignancies including work to suggest that levels of LMO1 may have a role in increased susceptibility to and increased aggressiveness of neuroblastoma.^13, 14^

In the developing mouse embryo, *Lmo1* is expressed in forebrain, hindbrain, the developing eye, developing olfactory system and spinal cord (EurEXPRESS^15^). *LMO1* expression in adult mouse tissues is most prominent in the bladder and a subset of neural tissues such as the retina and hippocampus (BIOGPS^16^). Cis-regulatory control mechanisms responsible for directing the expression of the *LMO1* paralogue *LMO2*, which also functions as a T-ALL oncogene, have been investigated in significant detail.^17, 18, 19^ However, our current understanding of the cis-regulatory control of *LMO1* expression is markedly lacking by comparison. Studies published to date have reported two alternative transcripts in cell lines by northern blotting,^2, 20^ which are the result of transcriptional initiation from two alternative promoters,^21^ one of which was shown to drive expression to the developing hindbrain.^22^ However, no distal regulatory elements nor upstream regulators have been identified so far. Moreover, the possible involvement of *LMO1* regulatory elements in mediating ectopic expression in T-ALL had not been explored.

Here, we used a combination of transgenic and transcriptional assays that allowed us to show that the *LMO1* promoters are primed for ectopic expression in T-cell leukaemias due to the presence of bivalent promoter histone marks and a latent haematopoietic enhancer. Following activation of the *LMO1* promoters and enhancer in T-ALL, the enhancer is bound in primary T-ALL cells by SCL and GATA3, thus suggesting that breakdown of epigenetic repression of *LMO1* represents a key step in the activation of a reinforcing loop of T-ALL oncogenes.

## Materials and methods

### Transgenic mouse analysis

Promoter and enhancer regions were PCR-amplified from human genomic DNA (promoter 1: hg19 chr11; 8290024-8290905, promoter 2: hg19 chr11;8284924-8285968), subcloned into a *lacZ* reporter vector and transgenic mice were generated and analysed as previously described^17^ A total of 35 transient transgenic mouse embryos were analysed. Selected embryos were cleared, sectioned, stained and photographed as previously described.^17^ All animal experiments were performed in accordance with UK Home Office rules and were approved by Home Office inspectors.

### Epigenomic data repository

NIH Roadmap Epigenomics data were accessed via www.roadmapepigenomics.org, including histone modification data for human ES cells, CD3 T-cells, CD109 B-cells and CD15 monocytes as well as DNaseI hypersensitivity in CD34 cells. ENCODE Project data were accessed via genome.ucsc.edu for transcription factor-binding sites and CTCF boundaries in K562 cells.

### Cell preparation and culture

Human T-ALL peripheral blood and bone marrow aspirate samples were obtained following informed consent at diagnosis from children and adults with T-ALL via a study protocol approved by the Research Ethics Committee of Addenbrooke's Hospital and the University of Cambridge. Banked T-ALL samples were recovered for 12 h in RPMI1640 supplemented with 20% FCS. Fresh T-ALL samples, X-ALLs CD3 and CD19 lymphocytes, CD34 cells, HUVECs and cultured megakaryocytes were prepared as described previously.^19^

### Real-time PCR estimation of total *LMO1* expression

RNA was prepared from patient samples and cell lines with Trizol reagent (Invitrogen, Paisley, UK). cDNA was prepared using random hexamers and TaqMan reverse transcriptase reagents kit (Applied Biosystems, Paisley, UK). Quantitative PCRs (qPCR) were run twice in triplicate using Stratagene Brilliant Sybr Green QPCR Master Mix (Agilent Technologies, Wokingham, UK) (Primers listed in Supplementary information). Standard curves for *LMO2* and *β*-actin were created by dilutions of CD4 cDNA. The data are reported as expression (normalised to β-actin housekeeper) relative to CD4. Error bars represent s.d.

### Chromatin Immunoprecipitation

Chromatin immunoprecipitation was carried out as described previously^23^ using 1 × 10^7^ cells per condition using commercially available antibodies (Supplementary Information). The relative enrichment of immunoprecipitated DNA was estimated using real-time PCR (Primers listed in Supplementary Information). Enrichment at each region was calculated relative to ChIP IgG pulldown and to the negative control region. Each reaction was undertaken twice and the experiment was undertaken on two occasions. The means of the data points are plotted with error bars representing s.d.

### Luciferase reporter assay

Plasmid DNA was prepared and the cells were transfected, subjected to antibiotic selection at an appropriate dose (as determined by kill-curve), lysate prepared and assayed as described previously.^17^ The mutant enhancer construct was generated by long PCR (using Del_LMO1enh forward and reverse primers; see Supplementary Materials for primer sequences). The PCR primers were designed to replace the conserved core region of the enhancer with an NheI restriction site. Data from four technical replicates of experiments designed with four biological replicates of each construct were normalised against pGL2 basic vector and background was set as 1. The mean of these data points is plotted with error bars representing s.d. Three biological replicates, each assayed in four technical replicates, were analysed for the enhancer deletion construct. Paired *t*-tests were used to determine the significance of loss of enhancer activity with the enhancer mutation, and made use of all 12 raw datapoints for each construct.

## Results

### The human *LMO1* gene locus contains two alternative promoters that recapitulate endogenous *LMO1* expression in transgenic mouse assays

**Figure 1:**
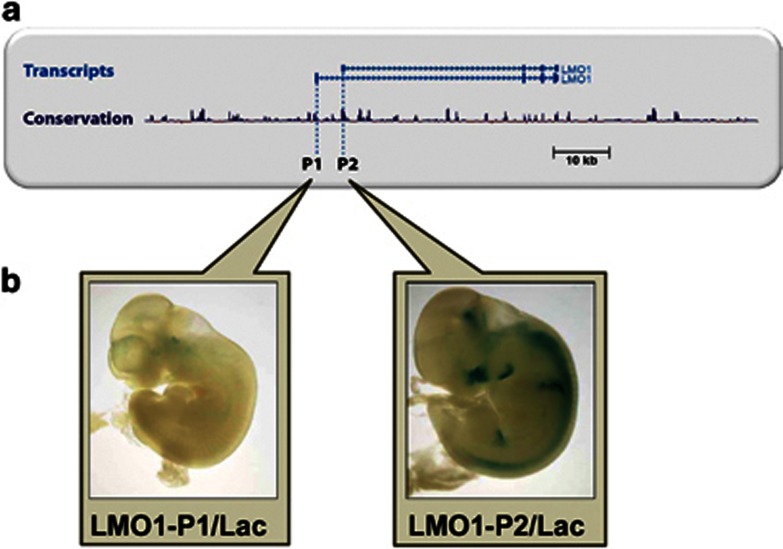
The human *LMO1* gene locus contains two alternative promoters that recapitulate endogenous *LMO1* expression in transgenic mouse assays. (**a**) Two alternative *LMO1*-validated transcripts are evident on review of the UCSC gene transcript database. Each coincides with a peak of non-coding sequence conservation suggesting possible roles as alternative promoters for *LMO1*. (**b**) Transgenic analysis of reporter constructs with the two respective promoter sequences upstream of a *lacZ* reporter gene. Representative embryos demonstrate that the promoter1/*lacZ* construct generated faint staining compatible with activity in neurological structures in the brainstem and first and second branchial arches. Transgenic embryos carrying the promoter2/*lacZ* construct showed expression in hindbrain, spinal cord, intersomitic mesoderm and olfactory epithelium thereby recapitulating much of the expression pattern found for endogenous *LMO1*. No *LMO1* expression is seen in tissues of the developing haematopoietic system in embryos generated by either construct.

The transcriptional control of *LMO1* has not been reported in any detail. We began our investigations by reviewing the RIKEN transcript database and database of transcriptional start sites (DBTSS) as well as ENSEMBL and UCSC genome annotations, and noted two alternative *LMO1* transcripts (Figure 1a). These alternative 5′ start sites coincide with peaks of non-coding sequence conservation suggesting possible roles as alternative promoters for *LMO1*. This is compatible with the work done previously,^20, 24^ where two alternative promoters of *LMO1* were proposed following northern hybridisation in the *LMO*1-translocated T-ALL cell line RPMI8402 and the human neuroectodermal small cell lung cancer-derived cell line N417. Following the mapping of the sequence data reported by Boehm *et al.*^21^ in 1991 to the human reference genome, we confirmed that the limited sequence data reported >20 years ago indeed corresponds to the two evolutionarily conserved start sites indicated in current annotations of the human genome.

To investigate the *in vivo* function of the two *LMO1* promoters, reporter constructs were generated (see Materials and Methods) with the two respective promoter sequences upstream of a *lacZ* reporter gene. Following pronuclear microinjection, F_0_ transgenic mice were generated, permitting the assessment of promoter activity by wholemount staining of E11.5 embryos. Representative embryos (Figure 1b) demonstrated that the promoter1/*lacZ* construct generated faint staining compatible with activity in neurological structures in the brainstem and first and second branchial arches. Transgenic embryos carrying the promoter2/*lacZ* construct showed expression in hindbrain, spinal cord, inter-somitic mesoderm and olfactory epithelium thereby recapitulating much of the expression pattern found for endogenous *LMO1* (Supplementary Figure S1). Taken together, these *in vivo* transgenic data allowed us to validate the two putative *LMO1* promoters as *bona fide* regulatory elements. The observed expression patterns correlated well with the known *LMO1* expression domain, and were also consistent with the lack of notable *LMO1* expression in tissues of the developing haematopoietic system.

### *LMO1* promoters display bivalent chromatin modification marks in a range of haematopoietic cells

**Figure 2:**
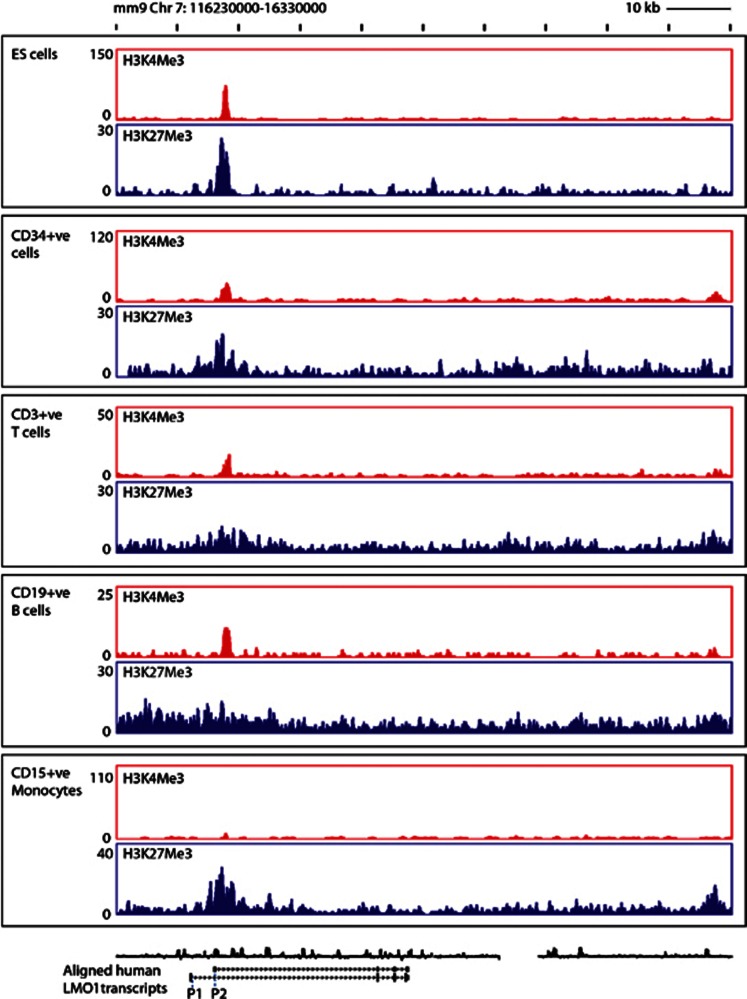
*LMO1* promoters display bivalent chromatin modification marks in a range of haematopoietic cells. Enrichment peaks are seen for both the activating H3K4Me3 and repressive H3K27Me3 histone mark in the 5' region of *LMO1* in ES cells, CD34 cells, CD3 T-cells and CD19 B–cells, with peak regions for both the activating and repressive histone marks over the promoter 2 (P2) region in these cell types. The positive H3K4me3 mark was absent in CD15 monocytes with only the repressive mark remaining.

Despite a clear role in leukaemia, there is no significant *LMO1* expression at major stages of normal haematopoietic differentiation (Supplementary Figure S2). To investigate whether lack of expression in the haematopoietic system may be due to active repression of the two *LMO1* promoters, we analysed ChIP-Sequencing data released to the public domain by the NIH Roadmap Epigenomics Mapping Project^25^ (http://www.roadmapepigenomics.org/). Remarkably, enrichment peaks for both the activating H3K4Me3 and repressive H3K27Me3 histone mark were present in the 5' region of *LMO1* in ES cells, CD34 cells, CD3 T cells and CD19 B cells (Figure 2). Peak regions for both the activating and repressive histone marks coincided and were situated at the promoter 2 (P2) region, thus marking this promoter as a bivalent promoter in these cell types. Of note, the positive H3K4me3 mark was absent in CD15 monocytes with only the repressive mark remaining. Lack of substantial expression was confirmed for all cell types by the absence of H3K36Me3 enrichment, (Supplementary Figure S3) in line with previously reported expression data.^6, 16^

Bivalent promoter marks in the absence of gene expression have traditionally been associated with genes being poised for expression.^26^ Given the potentially fatal consequences of expressing *LMO1* within the T-cell lineage, the poised status of *LMO1* promoters was unexpected. Importantly, inspection of the neighbouring genes confirmed that the absence of H3K36Me3 signal over the *LMO1* locus was not subject to technical failure (Supplementary Figure S4). Of note, bivalent promoter marks were also seen in primary mouse megakaryocytes in datasets generated as part of the mouse ENCODE consortium (see Supplementary Figure S5A). Taken together, this analysis suggests that, despite no known role for *LMO1* in normal haematopoietic cells, the gene is poised for expression in several haematopoietic lineages including T-lymphoid cells, which given its known function as a T-ALL oncogene is highly significant.

### High *LMO1* expression is evident in a significant proportion of T-ALL cell lines and patient samples

**Figure 3:**
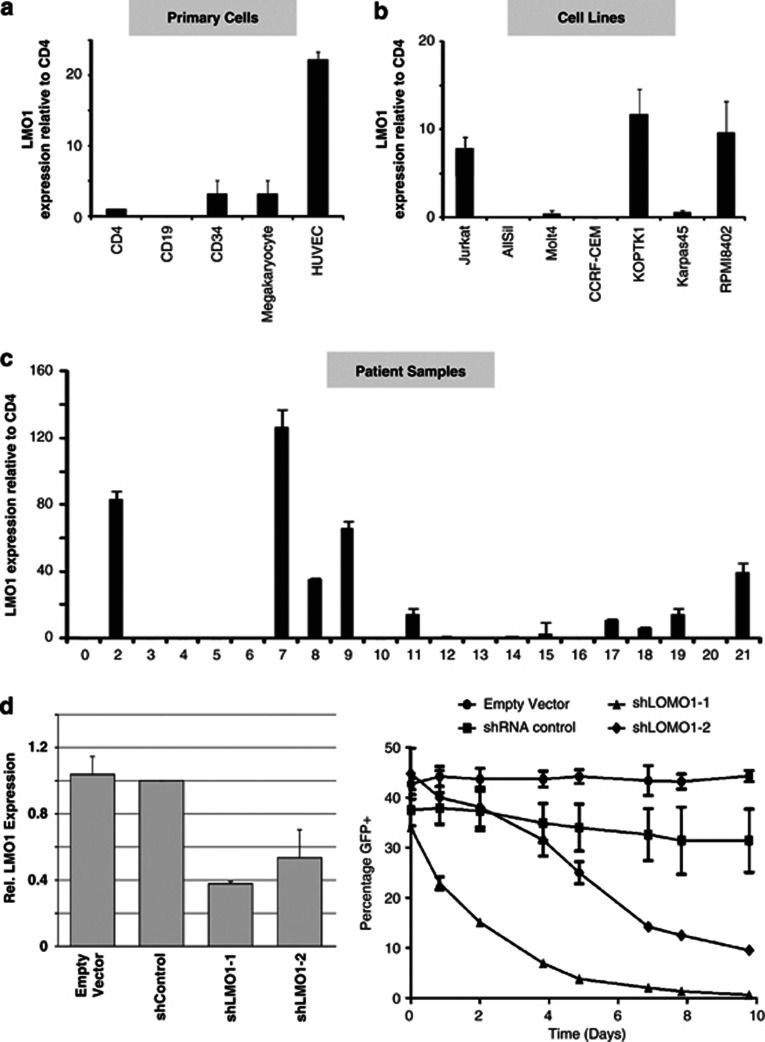
High *LMO1* expression is evident in a significant proportion of T-ALL cell lines and patient samples. (**a**) Very low level *LMO1* expression was observed in CD34 cells and in megakaryocytes at levels not significantly above those seen in CD4 T-cells. By contrast, markedly higher levels of expression were detected in cultured endothelial cells. (**b**) Three of the seven T-ALL cell lines (Jurkat, KOPTK1 and RPMI8402) had *LMO1* expression levels significantly above those in CD4 T-cells, two cell lines had similar *LMO1* expression levels to those seen in CD4 T-cells (Molt4 and Karpas45) with the remainder having undetectable levels of expression (AllSill and CCRF-CEM). (**c**) Nine of twenty-one primary T-ALL samples assayed showed high levels of *LMO1* expression with three (patients 2, 7 and 9) showing very high levels of *LMO1* expression. (**d**) *LMO1* expression is reduced by shRNA knockdown, leading to lower cell proliferation. Two shRNA *LMO1* knockdown vectors were designed using the RNAi Central webtool (http://katahdin.mssm.edu/siRNA/RNAi.cgi?type=shRNA) and cloned into the GFP-encoding pLL3.7 plasmid. Jurkat cells were transduced and both shRNA constructs (shLMO1-1 and shLMO1-2) showed marked reduction in *LMO1* expression compared with the negative control shRNA (shControl) and empty vector. Moreover, growth of shRNA-transduced cells was markedly reduced for cells transduced with shLMO1-1 and shLMO-2, compared with empty vector and the negative control. Data shown are from a representative biological replicate experiment performed in duplicate.

We next wanted to identify cellular models that would allow us to assess a potential contribution of *LMO1* regulatory elements to ectopic expression in T-ALL. To directly quantify *LMO1* transcripts in a range of malignant and non-malignant cell types, real-time PCR was undertaken on four normal haematopoietic cell types, cultured endothelial cells (HUVECs), seven T-ALL cell lines and 21 primary T-ALL samples. Individual results are presented relative to the very low signal obtained with the CD4 cell sample, (Figure 3) as described by Asnafi *et al.*^3^ As noted previously, *LMO1* is not noted to be expressed in blood cells when analysed by expression microarray technology but, by using this more sensitive method, we were able to detect low-level expression that would likely be below the detection threshold of microarray technology. Of note, this low-level expression was significantly below the levels seen in an *LMO1*-expressing neuroblastoma cell line (Supplementary Figure S6A), and would be consistent with our finding of bivalent promoter marks over the *LMO1* promoter in several haematopoietic lineages.

As seen in Figure 3a, very low level *LMO1* expression was observed in CD34 cells and in megakaryocytes at levels not significantly above those seen in CD4 T-cells. By contrast, markedly higher levels of expression were detected in cultured endothelial cells. Three of the seven T-ALL cell lines (Jurkat, KOPTK1 and RPMI8402) had *LMO1* expression levels significantly above those in CD4 T cells with the remainder having undetectable levels of expression (AllSill and CCRF-CEM) or similar levels to those seen in CD4 T cells (Molt4 and Karpas45) (Figure 3b). Of the 21T-ALL primary samples analysed, nine showed high levels of *LMO1* expression. Patients 2, 7 and 9 (Figure 3c) showed very high levels of *LMO1* expression. This proportion of *LMO1*-positive T-ALL samples is similar to that previously reported where a significant subset of patients were found to express high levels of *LMO1*, often with no evidence of a chromosomal translocation involving the *LMO1* locus.^3^ Moreover, *LMO1* knockdown in Jurkat cells severely compromises their proliferative capacity (Figure 3d) pointing to a non-redundant role for *LMO1* in leukaemia maintenance. The expression survey carried out here therefore provided both cell line and primary patient cellular models to further investigate the potential contribution of *LMO1* regulatory elements to ectopic expression in T-ALL.

### A conserved non-coding region 3' of *LMO1* displays active chromatin marks and transcription factor binding in haematopoietic cells

**Figure 4:**
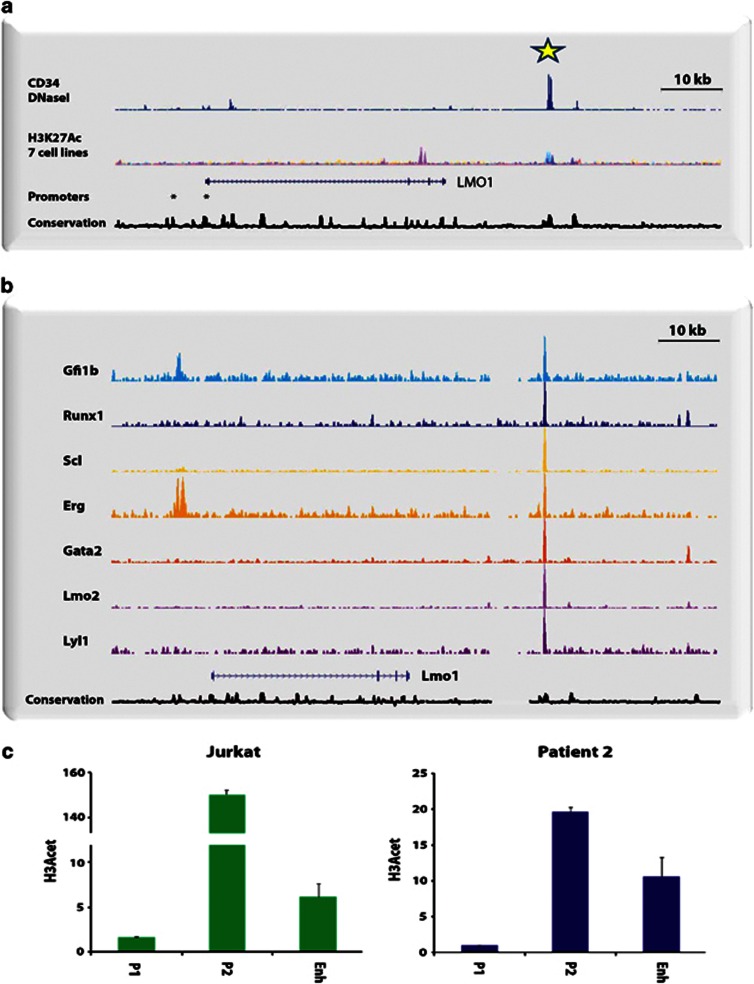
A conserved non-coding region 3' of *LMO1* displays active chromatin marks and transcription factor binding in haematopoietic cells: (**a**) A highly conserved region ∼20 kb 3' to the final exon of the human *LMO1* gene (+57 kb from the ATG immediately adjacent to the second exon) was identified in publicly available datasets from the ENCODE project, Roadmap Epigenomics project and UCSC goldenpath. This +57 region displayed marked DNaseI hypersensitivity, had histone H3 acetylation peaks in human umbilical vein endothelial cells (HUVEC) as well as the myeloid leukaemia cell line K562, and was bound by multiple transcription factors in a range of cell types. (**b**) Binding by multiple transcription factors including Gfi1b, Runx1, Gata2, Erg, Scl/Tal1, Lmo2 and Lyl1 to the mouse equivalent of the human *LMO1* +57 region in the HPC7 murine blood progenitor cell line. (**c**) Real-time PCR analysis of H3 Acetylation ChIP material demonstrated minimal enrichment at promoter 1, but significant enrichment at both promoter 2 and the +57 region, in a nontranslocated LMO1-expressing T-ALL (Jurkat) cell line and a non-translocated T-ALL patient sample.

Utilising publicly available datasets from the ENCODE project and Roadmap Epigenomics project, we noted a highly conserved region ∼20 kb 3' to the final exon of human *LMO1* (+57 kb from the ATG immediately adjacent to the second exon). This +57 region displayed marked DNaseI hypersensitivity, had histone H3 acetylation peaks in HUVEC cells as well as in the myeloid leukaemia cell line K562, and was bound by multiple transcription factors in a range of cell types (Figure 4a). Of note, this region was situated within regions bound constitutively by the genomic architectural protein CTCF either side of the *LMO1* gene locus, thus indicating that the +57 region lies within the likely boundaries of the *LMO1* regulatory domain (data not shown). To confirm transcription factor binding to this region in haematopoietic cells, we next investigated a previously published 10 transcription factor dataset for the HPC7 murine blood progenitor cell line.^27^ Again, we observed binding by multiple transcription factors including Gfi1b, Runx1, Gata2, Erg, Scl/Tal1, Lmo2 and Lyl1 to the mouse equivalent of the human *LMO1* +57 region (Figure 4b). The presence of active epigenetic marks (DNaseI and histone acetylation) together with the binding of multiple transcription factors therefore identified the +57 region as a candidate enhancer element. Moreover, as *SCL*, *LMO2* and *LYL1* have all been identified as T-ALL oncogenes in human patients^28, 29, 30^ and Erg has been shown to cause T-ALL in mouse models,^31^ binding of these factors in a myeloid progenitor cell line suggested that some of these factors may also act on the *LMO1* +57 enhancer in *LMO1*-expressing T-ALL cells.

We next undertook chromatin immunoprecipitation assays in nuclei generated from Jurkat, a nontranslocated *LMO1*-expressing T-ALL cell line as well as a nontranslocated *LMO1* expressing T-ALL patient sample using an antibody to acetylated histone H3, as this histone modification marks active promoters and enhancers (Figure 4c). Real-time PCR analysis demonstrated minimal enrichment at promoter 1, but significant enrichment was evident at both promoter 2 and the +57 region. Of note, enrichment levels were significantly lower in two primagrafts samples that did not express *LMO1* (see Supplementary Figure S5B). Taken together, these observations suggest that the *LMO1* +57 region functions as a transcriptional enhancer element in T-ALL cells, and may utilise known T-ALL oncogenic transcription factors such as *SCL* to mediate its activity.

### The *LMO1* +57 kb element directs reporter gene expression to haematopoietic tissues in F_0_ transgenic mice

Having established the *LMO1* +57 region as a DNA sequence carrying active epigenetic marks and bound by multiple transcription factors, we next addressed whether it can indeed function as a transcriptional enhancer. To this end, *lacZ* reporter constructs were generated by inserting the human +57 region downstream of a minimal promoter/lacZ reporter cassette, which would permit assessing the ability of the *LMO1* +57 kb region to direct expression *in vivo* in transgenic mice. Following pronuclear microinjection, F_0_ transgenic mice were generated and enhancer activity assessed by wholemount staining of E11.5 embryos. Seven out of eight transgenic embryos with lacZ staining showed activity in several neural tissues as well as in the heart and liver.

**Figure 5:**
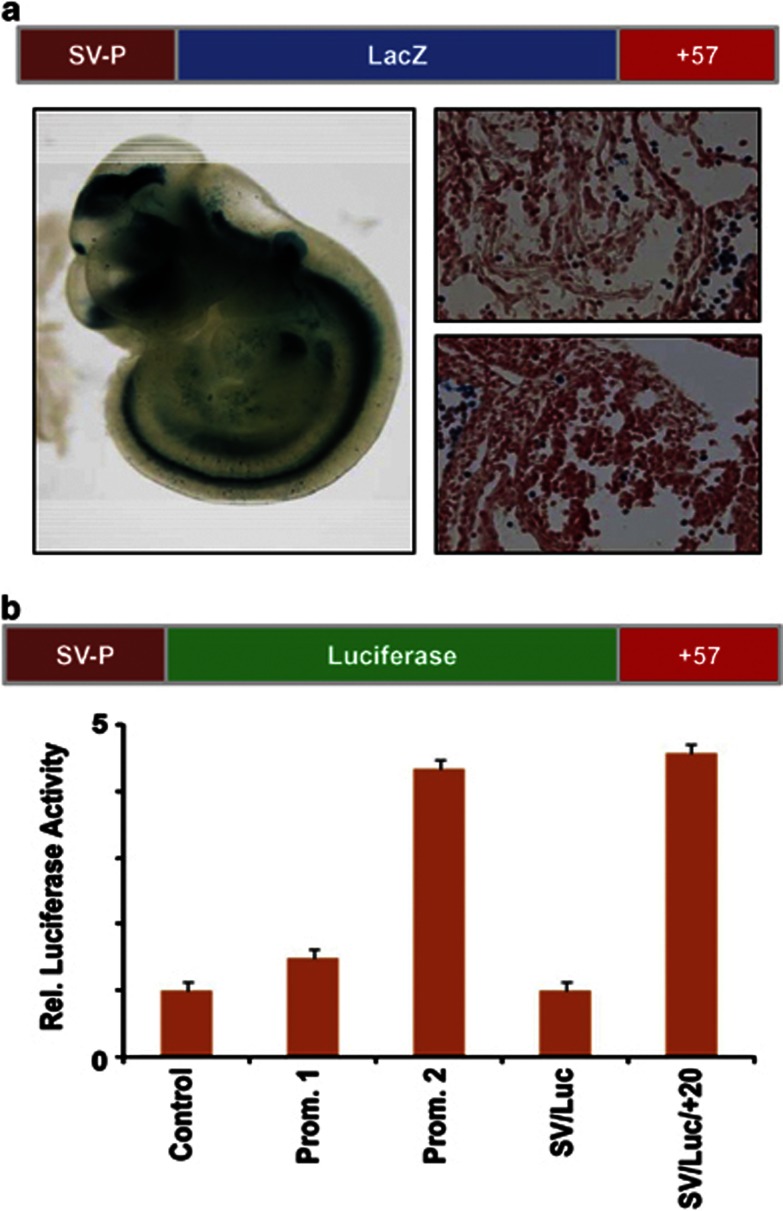
The *LMO1* +57 kb element functions as a transcriptional enhancer in blood cells. (**a**) F_0_ transgenic mouse analysis of the *LMO1* +57 region. Shown is a representative wholemount E11.5 embryo stained with X-Gal to determine the β-galactosidase expression pattern driven by the putative +57 enhancer. Histological sections of the same embryo confirm LacZ staining in circulating blood cells within the heart as well as the foetal liver. Wholemount images were taken at 2 × and tissue section images at 40 × magnification. (**b**) The promoter and enhancer elements are active in T-ALL cell lines. Luciferase reporter constructs were generated for the P1 and P2 promoter regions and the +57 putative enhancer region (LMO1 P1/luc, P2/luc and SV/luc/+57). These were stably transfected into *LMO1*-expressing, non-*LMO1*-translocated Jurkat cells alongside empty vector counterparts. All constructs showed greater activity than the empty vector (pGL2basic). Data shown are from three independent experiments, each performed in triplicate.

As evidenced by the representative embryo shown in Figure 5a, the *LMO1* +57 region indeed functions as a transcriptional enhancer element in transgenic mice, directing staining to multiple tissues including the eye, olfactory placodes, apical ectodermal ridge, intersomitic mesoderm, liver and heart. Importantly, *LMO1* +57 also directed expression to haematopoietic cells in the foetal liver (see histological sections in Figure 5a). Transgenic mouse assays therefore allowed us to validate the *LMO1* +57 region as a *bona fide* enhancer element with tissue-specific activity in transgenic mice that included developing haematopoietic cells within the midgestation embryo.

### The *LMO1* promoter and enhancer elements are active in T-ALL cell lines

Given that *LMO1* expression is minimal in normal blood cells but can be substantial in a subset of T-ALL leukaemia patients, we next investigated whether the *LMO1* promoters and +57 enhancer element showed activity in T-ALL cells. To this end, luciferase reporter constructs were generated using the same fragments of genomic DNA from the human *LMO1* locus that were used for the lacZ reporter constructs described in the previous sections. *LMO1* P1/*luc*, P2/*luc* and SV/*luc/* +57 were then stably transfected into *LMO1*-expressing, non-*LMO1*-translocated Jurkat cells alongside their empty vector counterparts. Following antibiotic selection, four independent pools of cells were assayed for each construct, and the whole experiment was repeated four times.

As shown in Figure 5b, both promoter elements showed greater activity than the control vector with promoter 1 demonstrating 1.5-fold higher activity than baseline and promoter 2 having 4.5-fold higher activity. Both these differences were statistically significant (*P*-values<0.01). Similarly, the enhancer construct SV/*luc/*+57 showed greater activity than the control vector (SV/luc), when assayed by stable transfection in Jurkat (*P*-values<0.01). Taken together, these results confirm that the +57 region not only functions as a haematopoietic enhancer in developing mouse embryos, but also displays transcriptional enhancer activity in a T-ALL transcriptional context.

### SCL/TAL1 and GATA3 bind to the *LMO1* +57 enhancer in T-ALL patient cells

**Figure 6:**
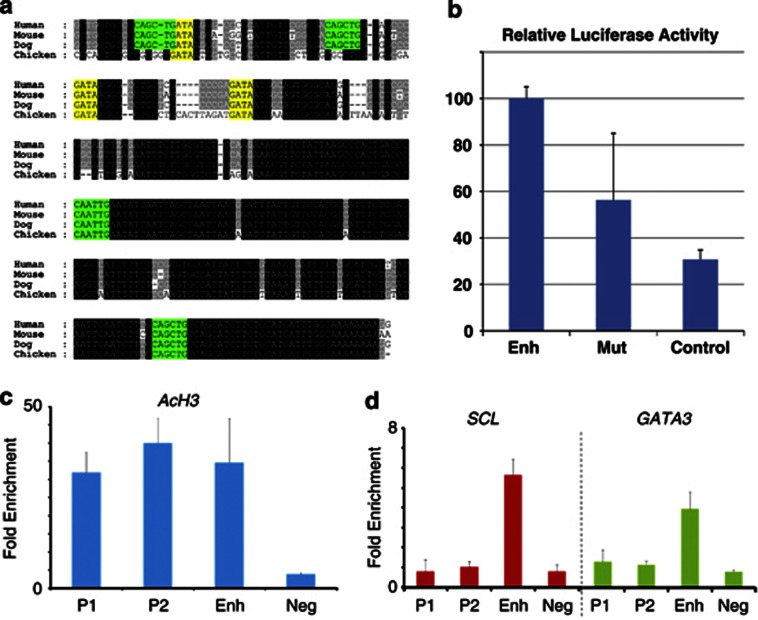
SCL/TAL1 and GATA3 bind to the *LMO1* +57 enhancer in T-ALL patient cells. (**a**) Nucleotide sequence alignment of the *LMO1* gene (+57 kb) enhancer region. Alignment of human, mouse, dog and chicken sequences extracted from the UCSC genome browser with conserved GATA (yellow) and E-Box (green) motifs coloured for clarity. Black boxes indicate 100% cross-species sequence conservation, grey boxes show less conserved sequences. (**b**) Stable transfection assays in Jurkat cells show that deletion of the region containing conserved E-Box and GATA motifs causes a significant reduction in activity of the +57 enhancer. Data from four technical replicates each of three biological replicates were normalised against the wild-type enhancer. The results show the mean of these data points with error bars representing s.d. Paired *t*-test analysis using all 12 raw data-points for each construct confirmed that the reduction in enhancer activity seen with the mutant enhancer was highly significant (*P*<0.01). (**c**) ChIP assays using an antibody against acetylated histone H3 (H3K9/K14) reveal elevated levels of histone acetylation at both *LMO1* promoters as well as the +57 enhancer region in an *LMO1*-expressing primagraft primary patient sample. (**d**) ChIP assays on T-ALL cells from the same primagraft (X31) using antibodies against SCL/TAL1and GATA3. Analysis by quantitative PCR demonstrates significant binding of both SCL/TAL1 and GATA3 to the *LMO1* +57 enhancer, with no binding to either of the two *LMO1* promoters.

Having demonstrated that the *LMO1* +57 enhancer is active in a *LMO1*-expressing T-ALL cell line, we next wanted to assess how its activating function may be mediated in *LMO1*-expressing T-ALL cells. To this end, we measured *LMO1* expression in six primary T-ALL xenografts^32^ passaged in immunodeficient mice (so called primagrafts). One sample (primagraft X31) showed high *LMO1* expression without any evidence of translocations involving the *LMO1* gene locus, and was therefore chosen for further analysis. Chromatin immunoprecipitation assays using an antibody against acetylated histone H3 (H3K9/K14) revealed elevated levels of histone acetylation at both *LMO1* promoters as well as the +57 enhancer region (Figure 6c). Given that histone acetylation is indicative of transcriptionally active promoters and enhancers, this observation suggested that the +57 enhancer is involved in mediating *LMO1* expression in T-ALL cells from primagraft X31.

As shown in Figure 6a, the central core sequence of the *LMO1* +57 enhancer is highly conserved between mammals, including consensus binding sites for transcription factors from the bHLH and GATA family (E-box and GATA motifs respectively). Moreover, deletion of the region containing conserved E-box and GATA motifs caused a significant reduction in enhancer activity (Figure 6b). Given that the leukaemogenic function of LMO1 is thought to require collaboration with bHLH factors such as SCL/TAL1 or LYL1.^8, 9, 33, 34^ The presence of the E-box motif within the +57 enhancer suggested the possibility of crosstalk between SCL/TAL1 and LMO1 through enhancing the activity of the *LMO1* +57 element. Moreover, Scl/Tal1 has previously been shown to interact with the T-lymphoid GATA factor GATA3 within T-ALL cells,^11, 35^ thus suggesting possible joint regulation of *LMO1* by SCL/TAL1 and GATA3 acting through the *LMO1* +57 enhancer. To assess possible involvement of SCL/TAL1 and GATA3, we performed ChIP assays using T-ALL cells from primagraft X31 using previously validated antibodies against SCL/TAL1^27^ and GATA3.^36^ (Figure 6d) Analysis of ChIP material by quantitative PCR demonstrated significant binding of both SCL/TAL1 and GATA3 to the *LMO1* +57 enhancer, but not to either of the two *LMO1* promoters. Moreover, Jurkat cells as well as our *LMO1*-expressing T-ALL primagraft sample showed high expression of *GATA3* and *SCL/TAL1*, and bioinformatic analysis of expression data for 92 T-ALL patient samples demonstrated that the TAL1 subcategory of patients showed the highest levels of *LMO1* expression (*P*=0.02; Mann–Whitney test) with *GATA3* expression high across all T-ALL subtypes (see Supplementary Figure S6B). Taken together therefore, these observations are consistent with a model whereby *LMO1* forms part of a core T-ALL regulatory circuit that involves other T-ALL oncogenes such as *SCL*/TAL1 and within which, the *LMO1* +57 enhancer has a central role integrating inputs from upstream regulators to mediate ectopic *LMO1* expression.

## Discussion

Dysregulation of transcription represents a common theme in human acute leukaemias including T-ALL.^37^ Molecular characterisation of translocation breakpoints demonstrated that in addition to the generation of fusion proteins with altered functionality, an alternative pathogenic event following translocation can be the juxtaposition of genes ordinarily not expressed into the vicinity of powerful regulatory elements, thus causing ectopic expression. The early paradigm for this mode of leukaemia oncogene function was provided by analysis of the t(8;14) translocation in Burkitt's lymphoma that results in the ectopic expression of *C-MYC*.^38^ Within T-ALL, the lim-only proteins *LMO1* and *LMO2* are similarly expressed ectopically as a consequence of translocations involving T-cell receptor gene loci.^24^ Importantly, mouse models have shown that ectopic expression of either *LMO1* or *LMO2* is sufficient to cause the development of T-ALL,^4, 39^ thus proving that out-of-context expression of native LMO proteins represents a leukaemogenic event during T-ALL development.

Recurrent translocations provide powerful proof of the oncogenic function of a particular gene product, as they would not be observed within a given disease unless they provide a clonal advantage. However, with a few exceptions, translocations involving an individual gene are only present in a minority of patients with a given type of leukaemia. Researchers have long argued that translocations involving a given gene may be suggestive of a wider role for this gene within a disease, extending to patients that do not carry the particular translocation. Particularly in the case of ectopic expression of the native protein, a multitude of mechanisms other than translocations could be considered as relevant underlying causes. Indeed, extensive surveys of T-ALL oncogene expression in patient samples demonstrated that for both *LMO1* and *LMO2*, the proportion of patients that express the oncogene vastly exceed the frequency of translocations.^3, 40^ In the case of *LMO2*, at least some of this excess expression has been explained by the demonstration of interstitial deletions removing a repressor element,^41^ and also by the realisation that a previously unrecognised 3rd *LMO2* promoter is active in T-ALL cells and bound by the ERG transcription factor, the ectopic expression of which can also cause T-ALL.^19^ By contrast however, potential mechanisms underlying ectopic expression of *LMO1* in the absence of translocations had not been explored.

Here, we have shown that the LMO1 gene locus is characterised by bivalent histone marks in multiple haematopoietic lineages including T-cells. Bivalent chromatin marks had originally been identified in ES cells, where simultaneous presence of the activating H3K4 and repressing H3K27 marks had been associated with promoters of many important lineage regulators, not expressed in pluripotent ES cells, but important for their differentiation into a range of different tissue types.^26^ Although the extent and biological implications of bivalency in ES cell chromatin biology have been challenged recently,^42^ the simultaneous observation of both activating and repressing histone marks within a population of cells not expressing the gene of interest is still likely to be indicative of a transcriptional status poised for expression. Given the potentially disastrous consequences of ectopic *LMO1* expression in the T-cell lineage, our observation of bivalent promoter marks in normal T-cells was particularly surprising.

With the somewhat paradoxical observation that a gene locus such as *LMO1* should be poised for expression in haematopoietic cells, the question arises as to whether there could be any biological reason for this potentially hazardous arrangement. Of interest in this context may be the recent report that single nucleotide polymorphisms within the *LMO1* locus carry significant associations with the risk of developing precursor B-cell leukaemia.^43^ Although the underlying mechanisms remain to be determined, it is possible that the increased risk of developing B-ALL is related to the expression levels of *LMO1*, particularly as none of the SNPs were coding and because SNPs affecting expression levels are thought to underlie the association of *LMO1* with neuroblastoma.^14^ Any model implicating expression levels would however posit that *LMO1* can be expressed in blood cells, in contrast to the current thinking based largely on northern blot and microarray analysis. Our more sensitive Q-reverse transcriptase-PCR analysis was able to detect low level expression of LMO1 in several haematopoietic lineages. These observations agree with recent comprehensive microarray surveys of both the mouse and human haematopoietic hierarchy, where low level *LMO1* expression can be observed in a small subset of haematopoietic cell types (see Supplementary Figure S7). Investigating any possible functions of LMO1 within normal haematopoietic cells should be the subject of future studies, and is likely to involve investigations into possible redundancy with other LMO proteins, in particular LMO2.

Ectopic expression accompanied by the resolution of bivalent promoter marks into activating marks only is consistent with biallelic expression of *LMO1* in nontranslocated T-ALL patients, in line with what has been shown before for *LMO2*.^40^ Biallelic expression not only focuses attention on the alterations within the leukaemic transcriptional environment that may be responsible for ectopically activating *LMO1*, but also the regulatory sequences within the gene locus through which such ectopic activation may occur. The presence of a highly conserved region downstream of *LMO1* that was bound by multiple transcription factors in haematopoietic cells prompted us to investigate the possible involvement of this genomic region in ectopic expression, particularly since several of the transcription factors bound to this region in stem/progenitor cells were known T-ALL leukaemia oncogenes (for example, SCL/TAL1 and LYL1). Indeed, we were able to show that this *LMO1* +57 region displays histone marks characteristic of transcriptional enhancers both in T-ALL cell lines and patient samples, and was bound by Scl/Tal1 and GATA3 in a primagraft T-ALL patient sample. Together with our demonstration that this region has haematopoietic activity when assayed in transgenic mice, this observation suggests that the *LMO1* +57 region may be involved in the low level *LMO1* expression observed in normal blood cells, and also become responsive to the altered transcriptional environment in a subset of T-ALL patients with the consequence of enhancing *LMO1* expression.

SCL/TAL1 and GATA3 had previously been shown to collaborate in mediating retinaldehyde dehydrogenase 2 expression in T-ALL cells,^11^ thus establishing this complex as a component of dysregulated transcriptional programmes in T-ALL. Our observation of SCL/TAL1 binding to the *LMO1* +57 enhancer now provides for the first time, evidence for a direct cross-regulatory link between SCL/TAL1 and LMO1, two leukaemia oncogenes known to collaborate during T-ALL development in mouse models.^7, 9^ A model is therefore emerging whereby high levels of SCL/TAL1 might contribute to the ectopic expression of LMO1, which in turn would serve to enhance the leukaemogenic function of SCL/TAL1 and thus provide a clonal advantage to SCL/TAL1-expressing preleukaemic T-ALL cells that activate expression of LMO1. Such a model then also focuses attention onto the molecular events that cause breakdown of the epigenetic processes that keep the *LMO1* locus repressed in normal haematopoietic cells, a better understanding of which would not only provide new insights into the pathogenesis of T-ALL, but may also open up new treatment opportunities, especially in light of the major current investment into the development of small molecule drugs targeting the epigenetic machinery.^44^
