# Supplementary material for: Bivalent promoter marks and a latent enhancer may prime the leukaemia oncogene LMO1 for ectopic expression in T-cell leukaemia
Source: Leukemia. 2013 Feb 1;27(6):1348–57. doi: 10.1038/leu.2013.2 (PMC3677138; doi:10.1038/leu.2013.2)
Supplement: Supplementary Information [file leu20132x1.pdf]

Supplementary information

Antibodies

GATA3	Upstate	sc22206x	1% crosslink
SCL	Upstate	sc12984x	0.4% crosslink
Acetylated Histone H3	Milipore	06-599	0.4% crosslink

Primers

cloning_LMO1P1_F	TAACTCGAGGGTAGCCACGGAGTCACATT
cloning_LMO1P1_R	TATAAGCTTAGCGCAGGAGGAGGGACT
cloning_LMO1P2_F	TAACTCGAGTGCCTTAGAACTGTGCTTGG
cloning_LMO1P2_R	TATAGATCTGCTTGTCCGGCTCTTAACCT
cloning_LMO1enh_F	TAAGGATCCCTCGCTCCAGCATTAGCAG
cloning_LMO1enh_R	TAAGTCGACCTTTGAGGGCTCAGGTTCC
Del_LMO1enh_F	TTAATTAGGCGGCCGCCTAATTAACGGATTTATTGTTC
Del_LMO1enh_R	GAGCACGCGCGGCCGCAGCCTCTCGGTAATTGATACAC
ChIP_RT_P1_F	TCTGGCTGTGCTTTTGTGTT
ChIP_RT_P1_R	GCCCGCCATTAGCTTATTTA
ChIP_RT_P2_F	AGGCTGAAAGCTGTGTCGTT
ChIP_RT_P2_R	ATGGCTCAATTTGCCCAGTA
ChIP_RT_enh_F	TTGTCTGAGGCTGTTTGCTC
ChIP_RT_enh_R	GAGTGTTAACCGCTGCCATT
ChIP_RT_neg_F	GAAATAAATATCTCCACTGTCCTG

ChIP_RT_neg_F	CTATCTGCCTATCTCTCATCTATC
LMO1_Expression_F	TCTACACCAAGGCCAACCTC
LMO1_Expression_R	AGCAGTCGAGGTGATACACG

Supplementary Figure legends

Figure S1

Images accessible via the EurExpress transcriptome atlas (www.eurexpress.org) show that LMO1 is expressed in the forebrain, hindbrain, spinal cord and inter-somitic mesoderm, olfactory epithelium and eye of E14.5 wild type murine embryos by RNA *in-situ* hybridisation. There is no staining of the liver or heart.

Figure S2

Data drawn from the BioGPS Gene Atlas expression profile repository (http://biogps.org) of LMO1 expression in normal human tissues demonstrates lack of expression of this gene in a diverse range of normal haematopoietic tissues. The informative 206718_at probe on the U133plus2 Affymetrix microarray platform was used. Data is displayed using z-scores produced by the barcode function of the R package "frma" http://www.bioconductor.org/packages/2.6/bioc/html/frma.html where a z-score >5 suggests that the gene is expressed in that tissue.

Figure S3

Data from the NIH Roadmap Epigenomics Mapping Project shows peaks of H3K4Me3 and H3K27Me3 in a region immediately upstream of the first exon of LMO1 in ES cells, CD34 cells, CD3 T cells and CD19 B cells and confirms lack of expression by absence of peaks of H3K36Me3in the region.

Figure S4

Data from the NIH Roadmap Epigenomics Mapping Project showing a wide-view of the LMO1 locus to demonstrate that data from H3K4Me3 and H3K27Me3 tracks has been scaled to maximum peak-heights in neighbouring genes to account for variation in read number. Additionally, the presence of regions of H3K36Me3 activity in neighbouring genes confirms that this data was not subject to technical failure.

Figure S5

Histone Modification Status in T-ALL Xenografts and mouse megakaryocutes. A) Mouse Encode ChIP-Seq data for H3K4me3 and H3K27me3 in primary mouse megakaryocytes. Shown is a screenshot of mouse ENCODE data from the Hardison lab (Penn State University) illustrating levels of H3K4me3 and H3K27me3 histone modification levels across the mouse LMO1 gene locus. Note that the LMO1 promoter regions carries bivalent marks, e.g. displays both H3K4 and H3K27 trimethylation. Data from the immunological genome project website show low level above threshold expression of mouse Lmo1 in a subset of monocyte/granulocyte cell types. B) Analysis of histone H3 acetylation by ChIP PCR. ChIP was carried out using an antibody against K9/K14 acetylated histone H3 on chromatin prepared from three T-ALL Xenografts with low (X27 and X30) as well as high (X31) LMO1 expression. ChIP material was anlaysed by Q-PCR using primer pairs specific for the LMO1 promoters P1 and P2, the LMO1 +52 enhancer region and also a negative control region from the LMO2 gene locus. When compared with the negative control region, it is clear that only Xenograft X31 showed substantial acetylation of the LMO1 enhancer region, consistent with this sample showing high LMO1 expression. The promoter P2 region showed some acetylation even in the very lowly expressing X27 and X30 Xenograft samples, consistent with the low level of acetylation seen in normal CD4 T-cells (see Figure 2).

Figure S6

Extended analysis of LMO1 expression. A) Comparison of LMO1 expression in leukaemic and non-leukaemic cells. LMO1 expression was analysed by Q-RT-PCR using RNA prepared from the neuroblastoma cell line SK-N-SH, the T-ALL cell line Jurkat and also CD4+ primary cells. Expression was analysed using SYBR Green, and normalised to levels of beta actin. The SK-N-SH cell line was used as a model for non-leukaemic expression of LMO1, as it has recently been used as a model for LMO1 expression in neural cells, the primary domain of LMO1 expression (see Nature. 2011 Jan 13;469(7329):216-20. ”Integrative genomics identifies LMO1 as a neuroblastoma oncogene”). B) Relative expression of LMO1, SCL/TAL1 and GATA3 in T-ALL samples. The three left-hand panels show LMO1, SCL/TAL1 and GATA3 expression in the 6 T-ALL xenografts as well as Jurkat cells used in this study. The panels on the right show expression of LMO1, SCL/TAL1 and GATA3 across a panel of 92 T-ALL patient samples, clustered based on their cytogenetic abnormalities and primary expression profiles (Blood. 2008 May 1;111(9):4668-80. “The recurrent SET-NUP214 fusion as a new HOXA activation mechanism in pediatric T-cell acute lymphoblastic leukemia”). It can be seen that Xenografts expressing LMO1 also express SCL/TAL1 and GATA3. Moreover, the TAL1 subcategory of T-ALL primary patient samples expresses the highest levels of LMO1 (P value using Mann-Whitney test is 0.02 (significant at 5% significance level) when comparing LMO1 expression levels in the Tal1 subcategory against all others); GATA3 expression is high across all T-ALL subcategories. -

Figure S7

Expression data from the Gene Expression Commons webtool (https://gexc.stanford.edu) show Lmo1 expression in a subset of mouse monocytes/granulocytes. The table below the expression colour-coded haematopoietic differentiation tree shows the actual expression values (log2 transformed microarray signal intensities).

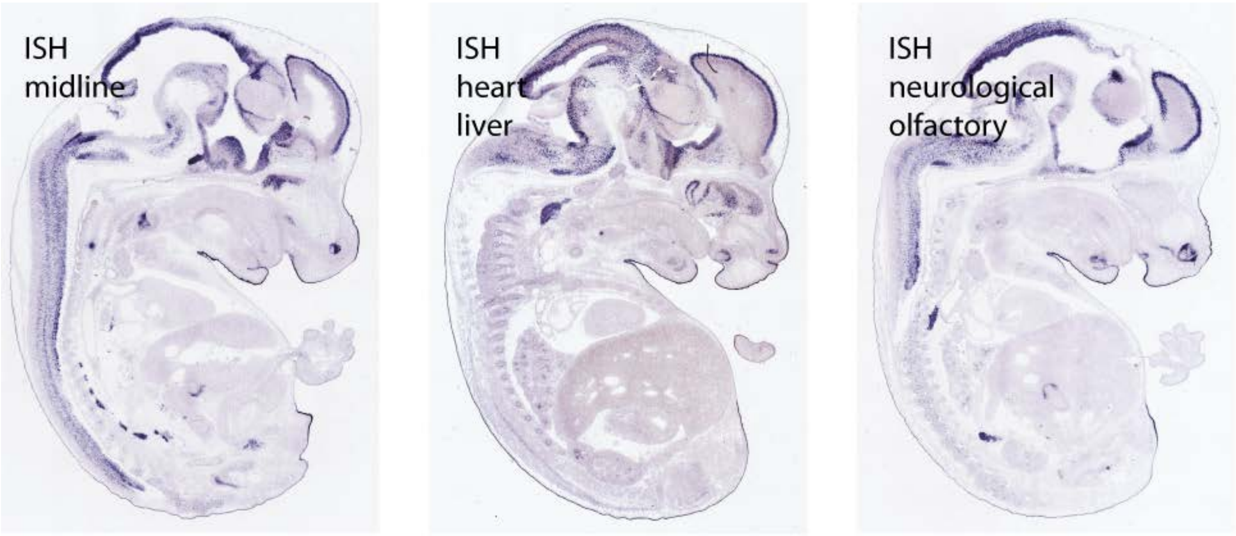

Figure S1

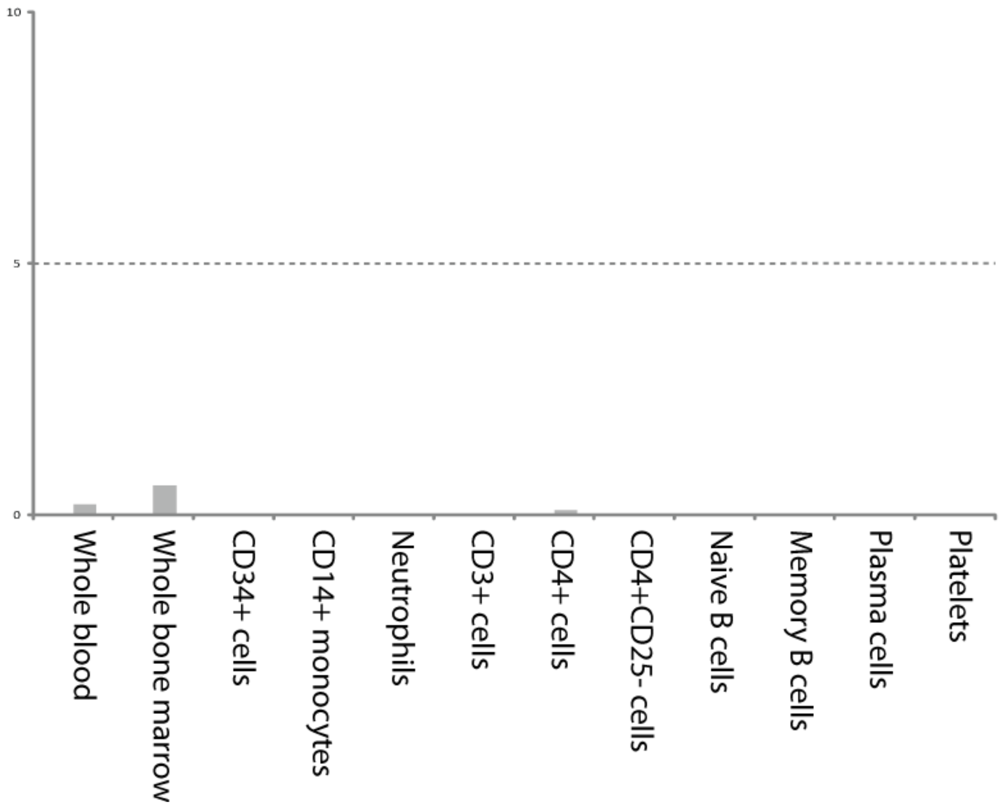
10
5
0
Whole blood
Whole bone marrow
CD34+ cells
CD14+ monocytes
Neutrophils
CD3+ cells
CD4+ cells
CD4+CD25- cells
Naive B cells
Memory B cells
Plasma cells
Platelets

Figure S2

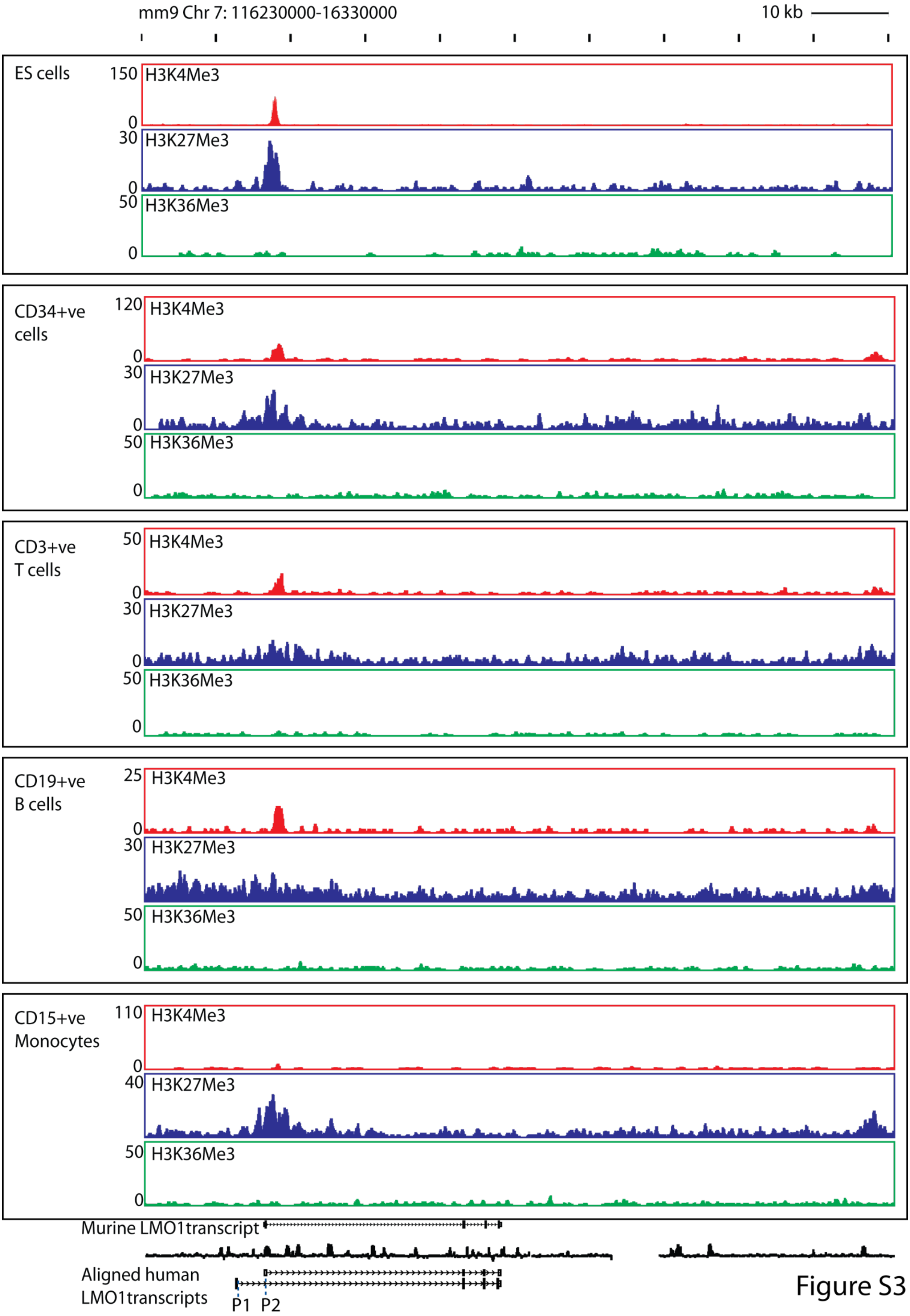
mm9 Chr 7: 116230000-16330000
10 kb
ES cells
150
H3K4Me3
0
30
H3K27Me3
0
50
H3K36Me3
0
CD34+ve cells
120
H3K4Me3
0
30
H3K27Me3
0
50
H3K36Me3
0
CD3+ve T cells
50
H3K4Me3
0
30
H3K27Me3
0
50
H3K36Me3
0
CD19+ve B cells
25
H3K4Me3
0
30
H3K27Me3
50
H3K36Me3
0
CD15+ve Monocytes
110
H3K4Me3
0
40
H3K27Me3
50
H3K36Me3
0
Murine LMO1transcript
Aligned human LMO1transcripts
P1
P2

Figure S3

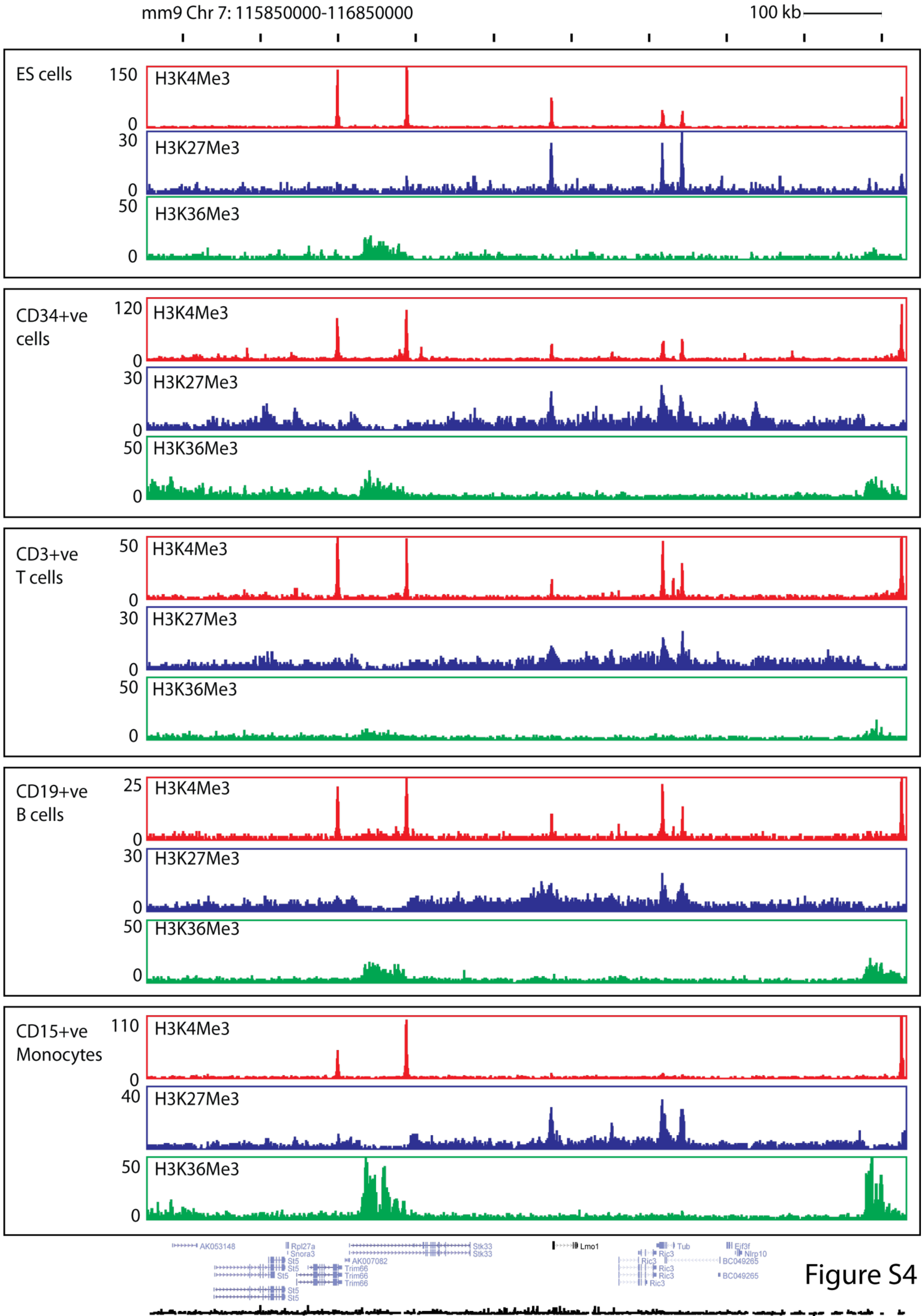
mm9 Chr 7: 115850000-116850000
100 kb
ES cells
150
0
30
0
50
0
H3K4Me3
H3K27Me3
H3K36Me3
CD34+ve cells
120
30
H3K4Me3
H3K27Me3
H3K36Me3
CD3+ve T cells
50
H3K4Me3
H3K27Me3
H3K36Me3
CD19+ve B cells
25
H3K4Me3
H3K27Me3
H3K36Me3
CD15+ve Monocytes
110
40
H3K4Me3
H3K27Me3
H3K36Me3
AK053148
Rpl27a
Snora3
St5
Stk33
AK007082
Trim66
Lmo1
Tub
Ric3
Eif3f
Nlrp10
BC049265

Figure S4

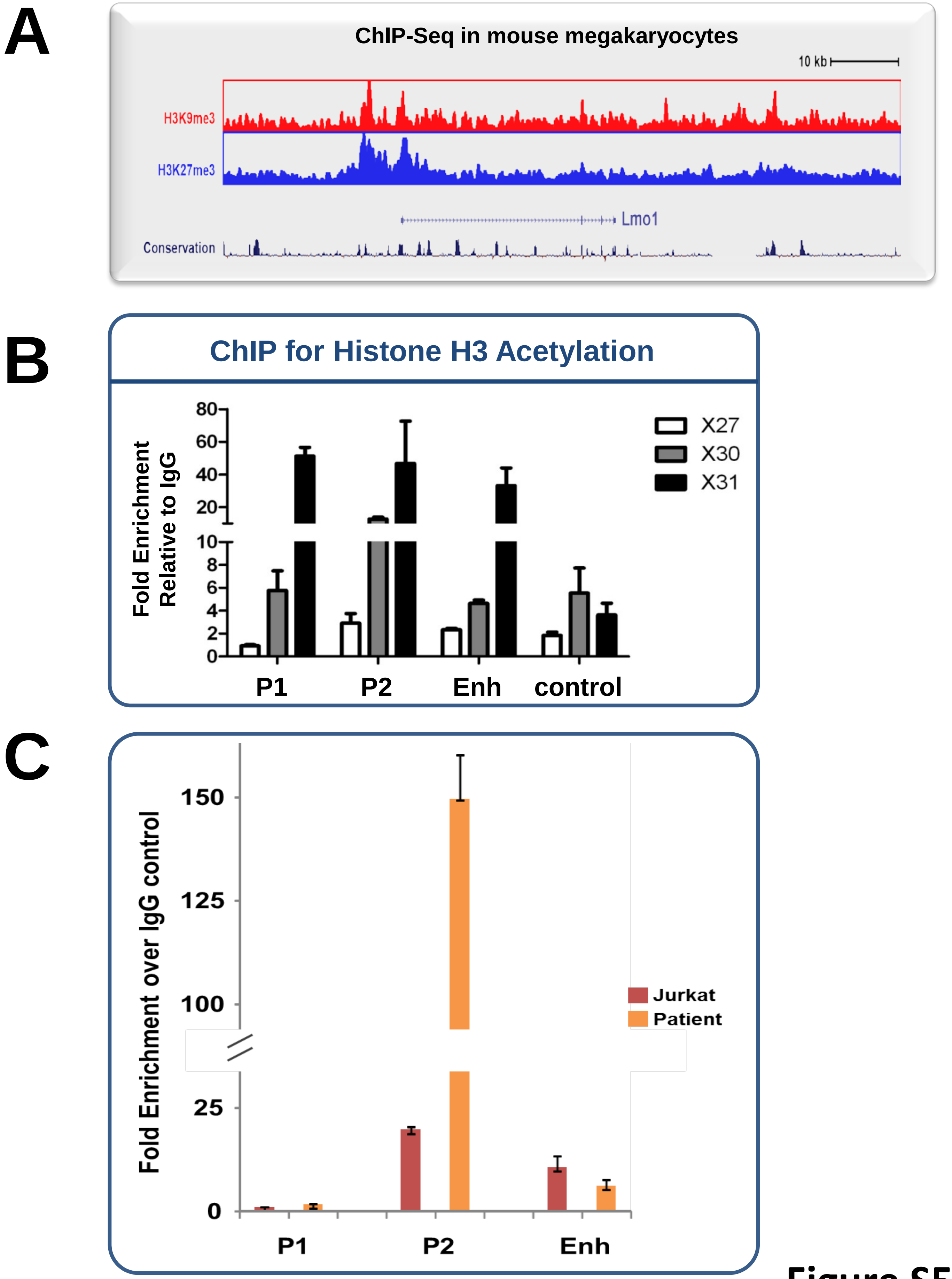
A
ChIP-Seq in mouse megakaryocytes
10 kb
H3K9me3
H3K27me3
Lmo1
Conservation
B
ChIP for Histone H3 Acetylation
Fold Enrichment Relative to IgG
X27
X30
X31
P1
P2
Enh
control
C
Fold Enrichment over IgG control
Jurkat
Patient
P1
P2
Enh

Figure S5

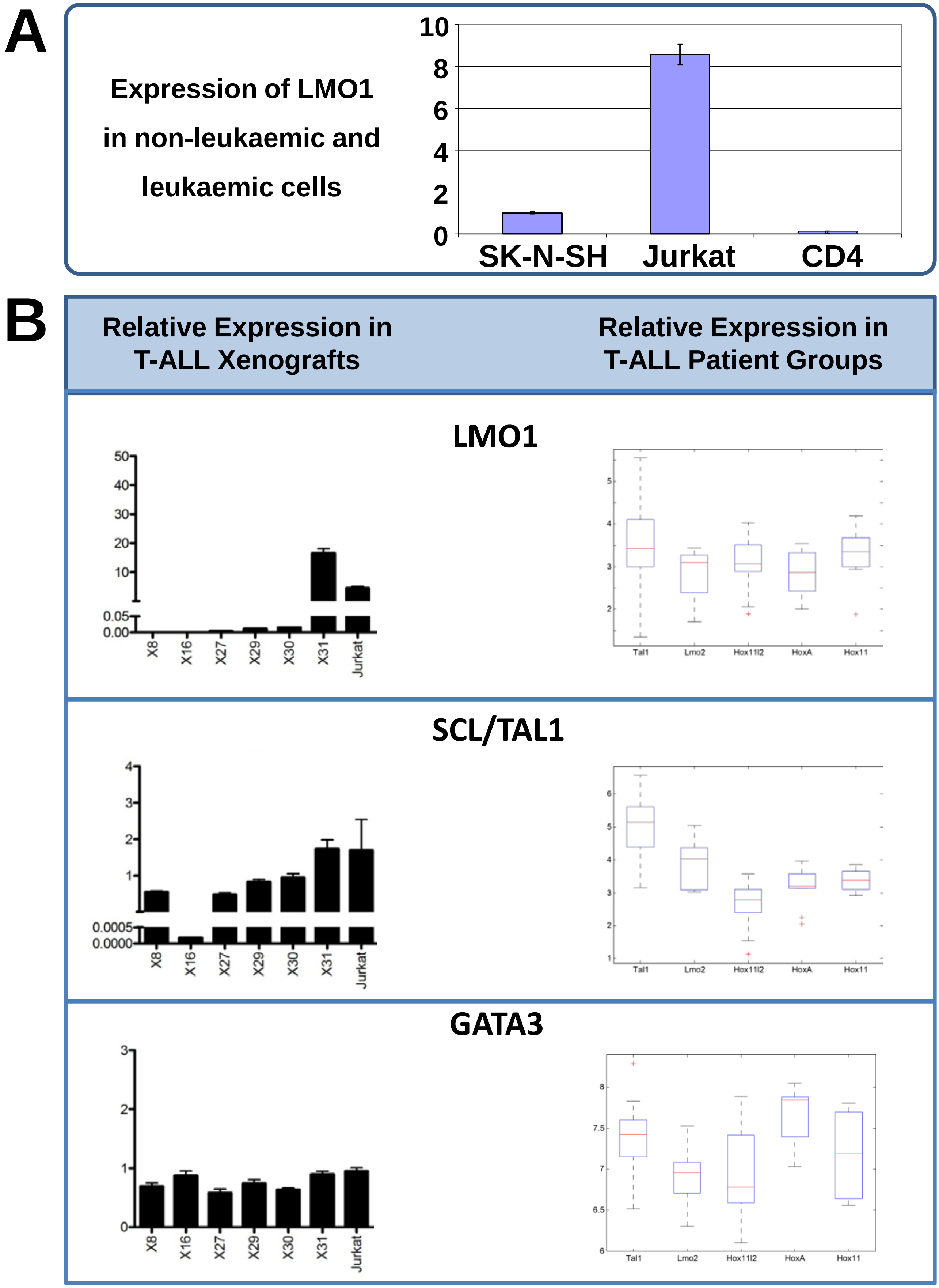
A
Expression of LMO1 in non-leukaemic and leukaemic cells
10
8
6
4
2
0
SK-N-SH
Jurkat
CD4
B
Relative Expression in T-ALL Xenografts
Relative Expression in T-ALL Patient Groups
LMO1
X8
X16
X27
X29
X30
X31
Jurkat
Tal1
Lmo2
Hox11l2
HoxA
Hox11
SCL/TAL1
GATA3

Figure S6

Gene Expression Commons

https://gexc.stanford.edu/

Gene Name Search Result

On **Mouse Hematopoiesis Model** submitted by Jun Seita (Stanford University)

Reference: PubMed ID: , GEO GSE ID: 34723

Lmo1 LIM domain only 1

Other Name: Rbtn-1, Rbtn1, and Ttg1

NCBI Entrez Gene: 109594

1 probeset was found.

Gene	Probeset	Alignment	Dynamic-range (99%)	Threshold
Lmo1	**1418478_at**	chr7:116282077-116314027 (-), 90.7, q31.2	7.04	6.76

1st Probeset: 1418478_at for Lmo1

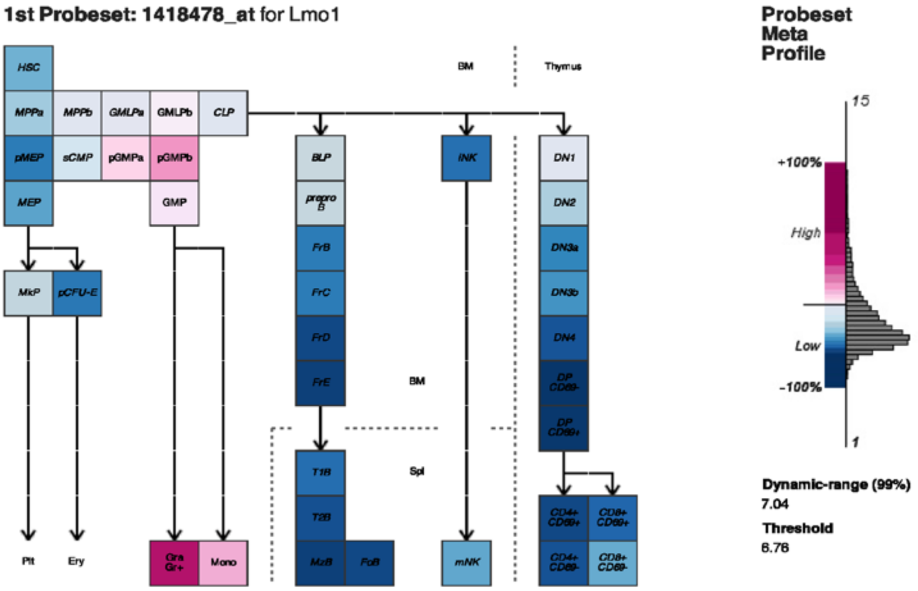

population	HSC	MPPa	MPPb	GMLPa	GMLPb	pMEP	sCMP	pGMPa	pGMPb	MEP	MkP	pCFU-E	GMP	Gra Gr+	Mono	CLP	BLP	prepro B	FrB	FrC
Average expression	5.54	5.72	6.55	6.68	6.89	5.17	6.15	7.12	7.74	5.41	5.97	5.13	7	9.62	7.49	6.52	6.02	6	5.17	5.16
Average percentile	-44.15	-34.35	-4.89	-1.61	10.69	-68.36	-17.14	26.12	51.95	-51.58	-23.34	-70.56	18.32	89.14	43.94	-5.6	-21.49	-22.35	-68.04	-68.66

Population	FrD	FrE	T1B	T2B	MzB	FoB	iNK	mNK	DN1	DN2	DN3a	DN3b	DN4	DP CD69-	DP CD69+	CD4+ CD69+	CD4+ CD69-	CD8+ CD69+	CD8+ CD69-
Average expression	4.83	4.73	5.06	4.93	4.73	4.84	5.07	5.45	6.57	5.81	5.24	5.3	4.97	4.53	4.58	4.85	4.95	4.99	5.48
Average percentile	-87.97	-91.91	-75.12	-83.15	-91.88	-87.74	-74.64	-49.16	-4.55	-30.22	-63.2	-59.09	-81.1	-96.18	-95.46	-87.13	-82.5	-79.61	-47.49

Figure S7
